# Resveratrol Supplementation Protects against Nicotine-Induced Kidney Injury

**DOI:** 10.3390/ijerph16224445

**Published:** 2019-11-12

**Authors:** Anand Ramalingam, Thulasiprevinnah Santhanathas, Shafreena Shaukat Ali, Satirah Zainalabidin

**Affiliations:** 1 Programme of Biomedical Science, Faculty of Health Sciences, Universiti Kebangsaan Malaysia, Jalan Raja Muda Abdul Aziz, Kuala Lumpur 50300, Malaysia; nate_anand@outlook.com (A.R.); thulasiprevinnah@moh.gov.my (T.S.); shafreenashaukatali@gmail.com (S.S.A.); 2 Institut Latihan Kementerian Kesihatan Malaysia, Jalan Pahang, Kuala Lumpur 50588, Malaysia

**Keywords:** antioxidant, irbesartan, nicotine, renal function, resveratrol

## Abstract

Prolonged exposure to nicotine accelerates onset and progression of renal diseases in habitual cigarette smokers. Exposure to nicotine, either via active or passive smoking is strongly shown to enhance renal oxidative stress and augment kidney failure in various animal models. In this study, we investigated the effects of resveratrol supplementation on nicotine-induced kidney injury and oxidative stress in a rat model. Male Sprague-Dawley rats were given nicotine (0.6 mg/kg, i.p.) alone or in combination with either resveratrol (8 mg/kg, i.p.), or angiotensin II type I receptor blocker, irbesartan (10 mg/kg, p.o.) for 28 days. Upon completion of treatment, kidneys were investigated for changes in structure, kidney injury markers and oxidative stress. Administration of nicotine alone for 28 days resulted in significant renal impairment as shown by marked increase in plasma creatinine, blood urea nitrogen (BUN) and oxidative stress. Co-administration with resveratrol however successfully attenuated these changes, with a concomitant increase in renal antioxidants such as glutathione similar to the conventionally used angiotensin II receptor blocker, irbesartan. These data altogether suggest that targeting renal oxidative stress with resveratrol could alleviate nicotine-induced renal injury. Antioxidants may be clinically important for management of renal function in habitual smokers.

## 1. Introduction

Smoking remains a major cause of chronic diseases worldwide. Smoking-related diseases alone contributed up to 6.4 million deaths worldwide in 2015, representing a 4.7% increase since 2005 [1]. In Malaysia, smoking-related diseases have been the primary cause of mortality for the past three decades. Smoking-related diseases were identified as a primary contributor to disability-adjusted life years (DALYs) measure of disease burden in Malaysia with ~20,000 deaths reported annually nationwide [2,3]. Although the government has introduced various campaigns, enacted smoke-free areas and raised taxes on tobacco products recently [4], high prevalence of smokers in the country is a major concern among researchers and professional in the health industry.

Long term exposure to nicotine, particularly via active or passive smoking has been linked to cardiovascular diseases and other chronic non-communicable diseases such as lung cancer [5,6]. However, the role of nicotine in promoting kidney diseases was only recognized in recent years [7,8,9,10]. Arany et al. has shown that sustained administration of nicotine for 28 days promotes p66shc-driven reactive oxygen species (ROS) production and oxidative stress in mice kidneys, and the nicotine intake in this mice model was relevant for chronic nicotine intake in human [7,8]. A more recent study also showed that prolonged nicotine administration for 30 days resulted in oxidative stress-mediated kidney damage in rat model [9]. Harwani et al. demonstrated that 14 days of nicotine infusion alone was sufficient to drive renal macrophage infiltration and premature hypertension in Wistar Kyoto rats and Spontaneously Hypertensive rats [10]. Nicotine-induced kidney injury may therefore play a role in development of chronic kidney diseases in smokers.

Although the mechanisms underlying nicotine-induced kidney injury remain poorly understood, past studies have collectively suggested that oxidative stress is a key mediator in nicotine-induced injury in kidney and other organs [7,9,11,12]. For these reasons, antioxidant supplements might be useful in slowing down or preventing nicotine-induced kidney injury among both active and passive smokers. Antioxidants may also be effective in reducing risk of chronic kidney disease among smokers; thereby potentially reducing smoking-related deaths. Indeed, antioxidants were proven to lower blood urea nitrogen and improve estimated glomerular filtration rate among patients with diabetic nephropathy [13]. End-stage renal disease also associated significantly with expression of superoxide dismutase (SOD) expression and total antioxidant status in patients undergoing dialysis [14].

Resveratrol (trans-3,4′,5-trihydroxystilbene) is a naturally occurring polyphenol found in red wine and pomegranates. It has been demonstrated to possess a wide range of pharmacological effects, including cardioprotection [15], neuroprotection [16] and protection against diabetic complications [17,18], as a result of its anti-inflammatory, antioxidant and cytoprotective properties. Previous studies have also demonstrated the benefits of resveratrol in several types of kidney diseases, including diabetic kidney disease [19], contrast-induced nephropathy [20], and sepsis induced kidney injuries [21]. It was also recently demonstrated that resveratrol attenuates nicotine-induced oxidative stress in renal proximal tubule cells in vitro via up-regulation of manganese-dependent superoxide dismutase (SOD) [22]. Despite all these evidences, it remains unknown whether resveratrol could prevent nicotine-induced kidney damage in vivo.

This study therefore was undertaken to investigate the effects of resveratrol supplementation on nicotine-induced kidney injury in a rat model. We demonstrate the effects of resveratrol supplementation on nicotine-induced changes in kidney injury markers, markers of oxidative stress as well as morphological changes in kidney after 28 days of administration, in comparison to the angiotensin II receptor blocker, irbesartan. Irbesartan is conventionally used for prevention and management of chronic kidney diseases.

## 2. Materials and Methods

### 2.1. Animals

Male Sprague-Dawley rats (5–6 weeks old, 180–230 g, *n* = 31) were obtained from Synertec Enterprise (Kuala Lumpur, Malaysia) and were housed under standard laboratory conditions in Universiti Kebangsaan Malaysia (UKM) Kuala Lumpur Campus Animal Facility for acclimatization. Standard rodent pellet and tap water were provided *ad libitum*. Each rat was inspected carefully for food and water intake, weight gain, signs of distress and injuries. Animals showing reduced weight gain, hunching, or bleeding injuries were all excluded from experiments. All procedures involving animals in this study adhered to the ethical guidelines provided by the UKM Animal Ethics Committee (UKMAEC) under the project code of FSK/BIOMED/2012/SATIRAH/12-DEC./486-DEC.-2012-DEC.2014.

### 2.2. Study Design

All rats were randomly allotted into four experimental groups, namely (1) vehicle control, (2) nicotine alone (NIC), nicotine plus resveratrol (NIC+R) and (3) nicotine plus irbesartan (NIC+IRB). Rats from all experimental groups except for the vehicle controls, were given 0.6 mg/kg nicotine dissolved in normal saline via intraperitoneal (ip) injection for 28 days as previously described [11]. Rats from NIC+R group also received 8 mg/kg resveratrol dissolved in DMSO vehicle for 28 days [23]. For rats from NIC+IRB group, rats were given 10 mg/kg irbesartan dissolved in DMSO vehicle in addition to nicotine for 28 days [24]. Vehicle control rats received saline vehicle and DMSO vehicle alone for the duration of this study. DMSO vehicle (5% *v*/*v*) was prepared using sterile distilled water. 

At study end, blood was collected from each animal via orbital sinus bleeding, for assessment of plasma cotinine using ELISA kit from Elabscience Biotechnology (Wuhan, China) and measurement of renal function markers. Kidneys were collected and used for analysis of kidney oxidative stress and histology.

### 2.3. Measurement of Blood Pressure

In vivo measurements of systolic blood pressure (SBP) and heart rate were obtained weekly via the non-invasive tail cuff method (CODA^TM^ non-invasive blood pressure system, Kent Scientific, USA) as previously described [25]. All rats were habituated to the CODA^TM^ system in a designated quiet room (27 ± 2 °C) for at least three consecutive days prior to acquisition of baseline measurements.

### 2.4. Measurement of Kidney Injury Markers

Plasma creatinine and blood urea nitrogen (BUN) were both determined spectrophotometrically as previously described [26]. 

For creatinine assay, plasma sample was added with 4 mL tungstic acid and centrifuged at 3000 rpm for 5 min. Supernatant was transferred into a 96-well microtitre plate and was added with picric acid and sodium hydroxide. The plate was incubated in the dark at room temperature for 15 min and colour development was measured at 520 nm wavelength. Plasma creatinine level was expressed as mg/dL based on standard curve generated using creatinine [27]. 

For estimation of BUN, plasma sample was added with isotonic solution and sodium hydroxide prior to centrifugation at 3500 rpm for 5 min. The supernatant was aliquoted and was added with diacetylmonoxime solution and phosphoric acid. The mixture was boiled at 100 °C for 30 min and the absorbance change was measured at 480 nm wavelength. BUN was expressed as mg/dL based on standard curve generated using urea [28].

### 2.5. Analysis of Kidney Oxidative Stress Markers

Kidney tissue samples were homogenized in chilled Tris-HCl pH 7.5 and were centrifuged at 12,000 rpm for 10 min at 4 °C. The supernatant was collected and used for measurement of superoxide dismutase (SOD) activity, reduced glutathione (GSH) level, and thiobarbituric acid-reactive substance (TBARS) level. All markers were normalized to total protein in kidney homogenate that was pre-determined using Bradford assay.

TBARS assay was used to detect malondialdehyde, the major lipid peroxidation product in the homogenate [29]. Kidney homogenate was reacted with thiobarbituric acid under acidic environment and boiled at 100 °C for 1 h. Pink colour development was measured after cooling down the mixture at 532 nm wavelength, and the TBARS concentration was expressed as mM/mg protein using a standard curve generated with 1,1,3,3-tetraethoxypropane.

GSH level in the kidney homogenate was measured using Ellman assay as previously described [30]. Kidney homogenate was mixed in Tris-HCl reaction buffer (pH 8.0) and was reacted with 5,5′-dithiobis-2-nitrobenzoic acid (DTNB) for 15 min in dark. Colour development after 15 min was measured at 412 nm wavelength; and the result was expressed as mmol/mg protein using a standard curve generated with GSH.

SOD activity was evaluated by its ability to inhibit the ferricytochrome reduction [31]. Kidney homogenate was mixed with assay mixture (containing phosphate buffered saline with EDTA, L-methionine, nitroblue tetrazolium, Triton-X and riboflavin) and was incubated in an aluminum foil-covered box under 20 Watt lamp for 7 min. Colour development was measured at 560 nm wavelength, and the specific activity of SOD was expressed as units of enzyme/min/mg protein, with one unit of enzyme inhibiting 50% of colour development. 

### 2.6. Kidney Histology

Kidneys excised from the rats were fixed in 10% neutral-buffered formalin, processed in a graded series of alcohol (50%, 70%, 80%, 90% and 100%), cleared in two changes of xylene and were embedded in paraffin. Paraffin sections of each kidney (2–3 µm) were then stained with Hematoxylin and Eosin (H&E), Masson Trichrome or Periodic Acid Schiff’s staining for observation of general kidney morphology, fibrosis and mesangial matrix expansion respectively [26,32].

### 2.7. Statistical Analysis

All data are presented as mean ± standard error of mean (SEM). One-way or two-way analysis of variance (ANOVA) followed by a Tukey’s post-hoc test was used to analyse differences between groups, unless otherwise mentioned. Statistical significance was considered at *p* < 0.05.

## 3. Results

### 3.1. Systemic Characteristics

All treatments had no effect on the final body weight of rats, however nicotine administration for 28 days significantly increased systolic blood pressure compared to vehicle controls (*p* < 0.05, Table 1). Resveratrol co-administration markedly prevented nicotine-induced increase in systolic blood pressure, similar to irbesartan-administered rats (both *p* < 0.05). Based on the plasma cotinine analysis, it was noted that nicotine intake among all three groups, i.e., NIC, NIC+R and NIC+IRB was comparable. No cotinine was however detected in the blood plasma of vehicle controls.

### 3.2. Kidney Injury Markers

**Figure 1:**
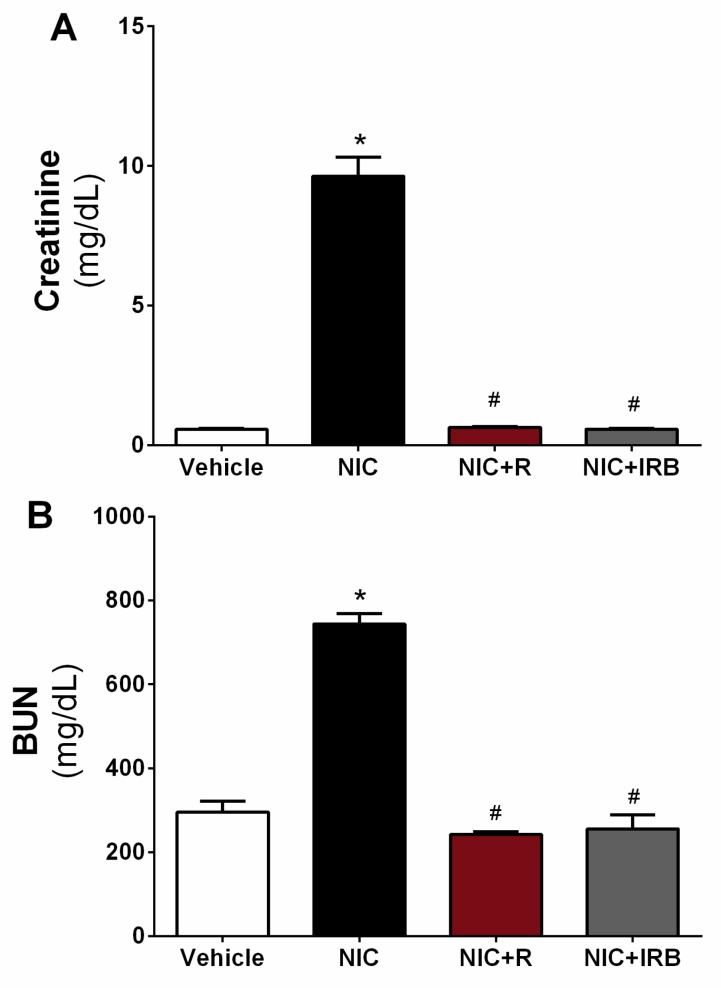
Effect of resveratrol supplementation on changes in plasma level of (**A**) creatinine and (**B**) blood urea nitrogen (BUN) after 28 days of nicotine administration. * *p* < 0.05 vs. vehicle and # *p* < 0.05 vs. NIC group using Tukey post-hoc test.

Figure 1A shows the estimation of creatinine level in plasma samples of all groups. Level of creatinine was increased significantly in the nicotine group compared to the vehicle controls (*p* < 0.05). A similar trend was seen for BUN estimation (as shown in Figure 1B) (*p* < 0.05 vs. vehicle controls). Conversely, plasma creatinine and BUN level were significantly lowered in NIC+R group as compared the NIC group (both *p* < 0.05). Irbesartan administration also markedly lowered plasma creatinine and BUN level compared to the NIC group (*p* < 0.05).

### 3.3. Oxidative Stress

**Figure 2:**
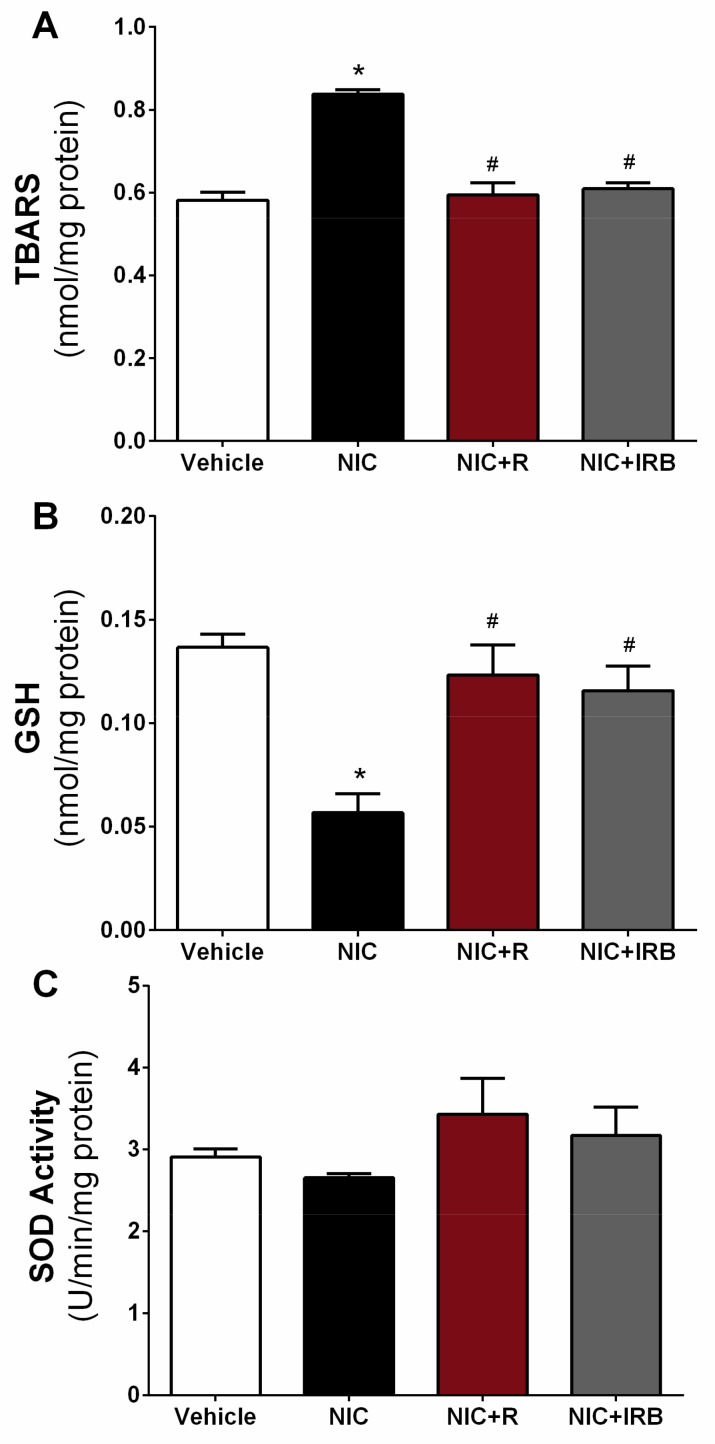
Effect of resveratrol supplementation on changes in (**A**) TBARS level, (**B**) GSH level and (**C**) SOD activity in rat kidneys after 28 days of nicotine administration. * *p* < 0.05 vs. vehicle and # *p* < 0.05 vs. NIC group using Tukey post-hoc test.

Figure 2A shows TBARS level in the kidney homogenate of all groups. Compared to the vehicle controls, TBARS level in the nicotine group was significantly increased after 28 days of administration (*p* < 0.05). Co-administration of resveratrol and irbesartan however significantly prevented such increase in TBARS level, as compared to the nicotine alone group (both *p* < 0.05). Increment in TBARS level among nicotine-administered rats was accompanied by concomitant reduction in GSH level (Figure 2B). Both resveratrol and irbesartan similarly increased GSH level in kidney homogenate as compared to the nicotine alone group (both *p* < 0.05, Figure 2B). Despite the increase in TBARS level and concomitant reduction in GSH level, total SOD activity was unaffected by the 28 days of nicotine administration in this study. As shown in Figure 2C, no significant difference was observed in SOD activity between all the experimental groups.

### 3.4. Kidney Histology

**Figure 3:**
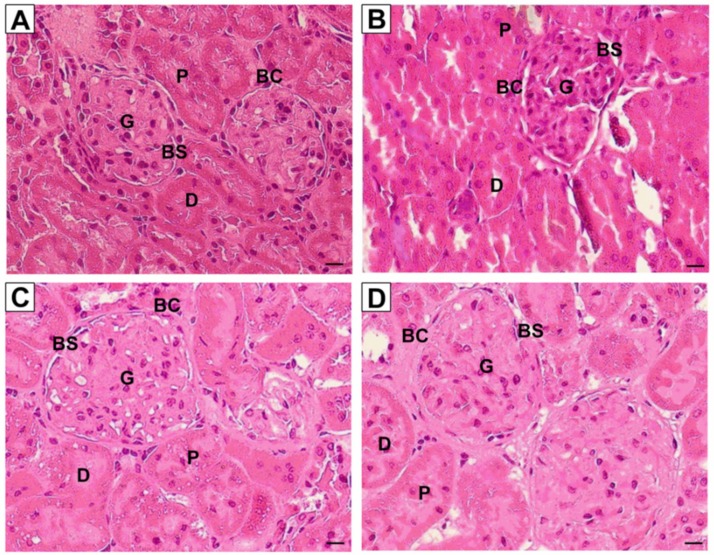
Representative images of kidney sections stained with routine Haematoxylin and Eosin (40× magnification, scale bar = 20 μm) for (**A**) Vehicle, (**B**) NIC, (**C**) NIC+R and (**D**) NIC+IRB groups respectively. G, glomerulus; BC, Bowman’s capsule; BS, Bowman’s space; P, proximal tubule; D, distal tubule.

**Figure 4:**
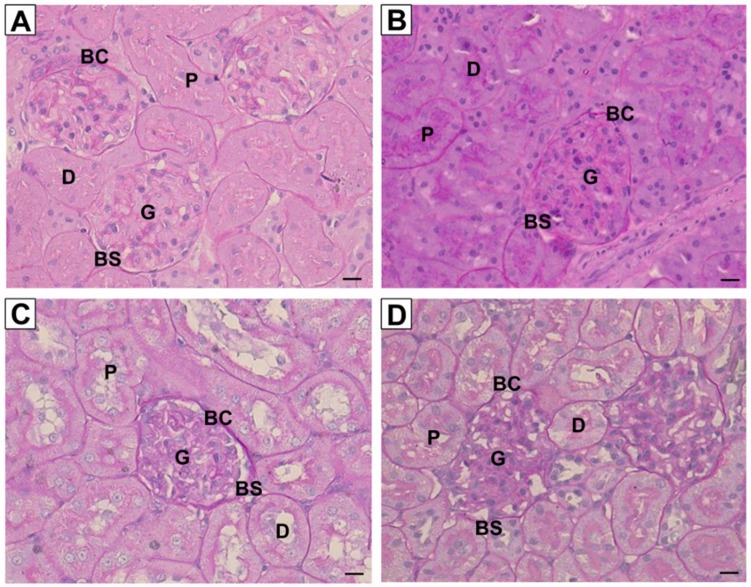
Representative images of kidney sections stained with PAS staining (40× magnification, scale bar = 20 μm) for (**A**) Vehicle, (**B**) NIC, (**C**) NIC+R and (**D**) NIC+IRB groups respectively. G, glomerulus; BC, Bowman’s capsule; BS, Bowman’s space; P, proximal tubule; D, distal tubule.

**Figure 5:**
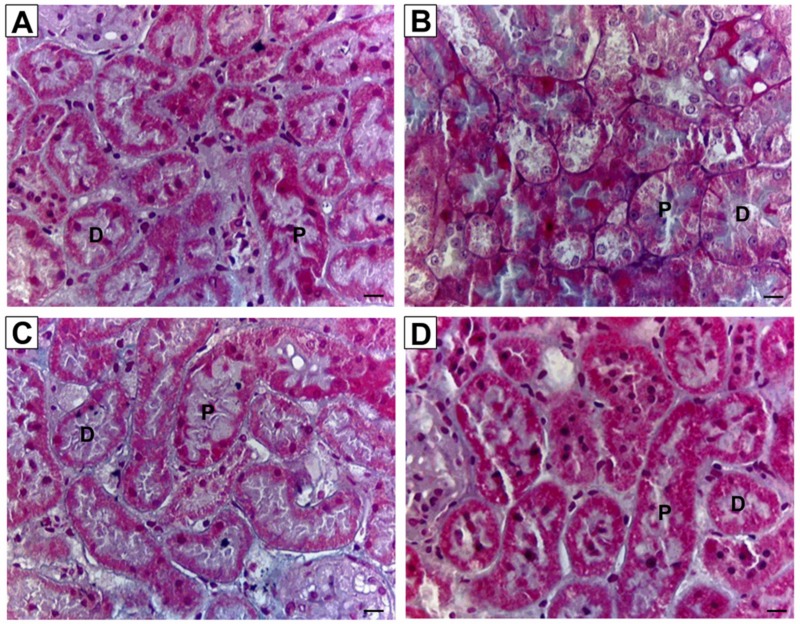
Representative images of kidney sections stained with Masson’s trichrome staining (40× magnification, scale bar = 20 μm) for (**A**) Vehicle, (**B**) NIC, (**C**) NIC+R and (**D**) NIC+IRB groups respectively. G, glomerulus; BC, Bowman’s capsule; BS, Bowman’s space; P, proximal tubule; D, distal tubule.

Figure 3 shows the representative images of kidney sections stained with routine hematoxylin and eosin from all experimental groups. For kidneys in the control group, no prominent damage was seen in the glomerulus, epithelial cells of Bowman’s capsule, as well as lumen brush border of proximal and distal tubules. Unlike the control group, kidneys in nicotine group showed damage to the epithelial lining in the nicotine group but the overall morphology was unaffected. Normal morphology of kidneys was observed for both NIC+R and NIC+IRB groups. Figure 4 shows the representative images of kidney sections stained with periodic acid Schiff’s (PAS staining). Although the intensity of PAS staining was increased in nicotine group; no signs of glomerulosclerosis or mesangial matrix expansion were observed among all four groups. Similarly, collagen deposition was not observed among all four groups, as shown by the kidney sections stained with Masson’s trichrome (Figure 5). This suggests that 28 days of nicotine administration did not cause glomerulosclerosis or renal fibrosis.

## 4. Discussion

This study demonstrated the effects of resveratrol supplementation on nicotine-induced kidney injury in vivo. In this study, we observed that 28 days of nicotine administration markedly increased plasma level of kidney injury markers and oxidative stress without causing overt irreversible damage to the kidney structure in a rat model. Resveratrol supplementation effectively blunted nicotine-induced oxidative stress and prevented nicotine-induced increases in kidney injury markers. Resveratrol-mediated protection was comparable to a conventional angiotensin II receptor blocker, irbesartan.

Plasma creatinine and BUN are primary indicators of kidney injury in rodents and humans. Increased creatinine and BUN is associated with poor glomerular filtration rate (GFR) and clearance of metabolic wastes from the body. Nicotine administration for 28 days significantly increased plasma creatinine and BUN as compared to the vehicle controls, consistent with several previous studies [33,34]. Although GFR was not directly measured in this study, it is possible that nicotine reduces GFR and causes retention of the creatinine and urea in plasma [35]. Resveratrol co-administration with nicotine significantly prevented the elevation in these renal injury markers. Resveratrol given to cisplastin-intoxicated animals attenuated the increase in serum creatinine and urea by inhibiting death receptor-mediated apoptosis [36]. Pre-treatment with 10 mg/kg resveratrol also reduced the mortality in rats subjected to renal ischaemia-reperfusion injury with a significant reduction in serum creatinine [37]. In this study, resveratrol-mediated reduction in kidney injury markers was comparable to those seen with irbesartan-administered rats; suggesting that resveratrol is highly effective in preventing onset of kidney injury similar to a conventional drug.

Kidney injury is frequently associated with oxidative stress. Up-regulation in ROS production together with concomitant reduction in endogenous antioxidants not only promotes damage to the glomerulus and tubular cells, but also drives fibrosis and inflammation seen in chronic kidney diseases [32]. In this study, TBARS level indicative of lipid peroxidation was significantly increased in the nicotine group as compared to the vehicle controls. This was accompanied by a concomitant reduction in GSH level in the kidneys; both together suggesting an increased oxidative stress after 28 days of nicotine administration. This observation was similar to several past studies [34,38]. Compared to the nicotine group, resveratrol administration significantly attenuated both markers of oxidative stress similar to irbesartan. Increased ROS production may promote peroxidation of lipid membrane in kidneys and cause increment in TBARS. GSH is one of the most important non-enzymatic antioxidants that protects membrane lipids from oxidation and maintain redox status in various cells. Several reports indicated that, tissue injury induced by various stimuli is coupled with GSH depletion [11,39]. We and others have previously shown that prolonged nicotine administration increases ROS and causes depletion of GSH in aorta and heart [40,41]. Erat et al. also showed that nicotine inhibits activity of glutathione reductase, thus preventing reduction of oxidized glutathione disulphide (GSSG) to GSH.

Resveratrol is a strong lipophilic antioxidant and is therefore very effective in lowering down lipid peroxidation and preserving endogenous antioxidants. Csiszar et al. [42] have previously demonstrated that resveratrol abrogated smoking-induced upregulation of ROS and inflammatory markers to protect aortic endothelial cells from apoptotic cell death. Similarly, in another study, resveratrol also significantly lowered level of lipid peroxidation markers such as TBARS and 4-hydroxynonenal after cisplatin cytotoxicity in rat renal cortical slices in vitro [43]. Consistent with these past observations, our findings suggest that resveratrol is capable of preventing lipid peroxidation in settings of kidney injury.

Interestingly, total activity of SOD was unaffected by all treatments in this group. Although past studies have shown that nicotine reduces gene and protein expression of manganese SOD in kidneys and tubular cells [8,22]; nicotine administration for 28 days failed to significantly reduce total SOD activity in rat kidneys. It is possible that the increase in total SOD activity in nicotine administered rats is a compensatory response to the oxidative stress and thus no difference was seen between all groups. Manganese SOD activity or expression was not measured in this study; thus it is not known whether nicotine selectively affected manganese SOD alone instead of total SOD activity. Future studies should therefore measure activities and expression of SOD isoforms to verify whether nicotine specifically inhibit specific isoform of SOD.

On histological analysis, kidney stained with H&E did not show significant damage to kidney structure in nicotine group. Toklu et al. [33] showed that nicotine administration for 21 days resulted in extensive degeneration with severe vasocongestion in the parenchyma, wide dilatations around the glomeruli, and vacuolizations and debris in the tubules. Such abnormal histology was not observed in rats from our study although nicotine-administered rat kidneys showed epithelial lining damage. Resveratrol and irbesartan both prevented epithelial damage seen in nicotine group. Resveratrol-mediated reduction in oxidative stress may have prevented structural damage to the kidneys; considering oxidative stress is a primary mediator of renal injury and glomerulosclerosis [32]. Nevertheless, we observed that nicotine administration for 28 days failed to induce glomerulosclerosis or renal fibrosis as shown by PAS and Masson’s trichrome staining. We speculate that a longer duration of nicotine administration is required to induce glomerulosclerosis or fibrosis, which are both characteristics of end-stage chronic kidney disease. Future studies may be designed to investigate the effects of resveratrol administration on these attributes in a more clinically relevant chronic nicotine model.

In the present study, it was shown that prolonged administration of nicotine in rats resulted in a plasma cotinine level similar to those found in chronic smokers (100–300 ng/mL) [44]. Other studies [38,44] have found that an exposure period of at least 2–3 week is necessary to consistently reach these levels. Therefore, nicotine intake in this model is comparable to chronic nicotine inhalation seen in smokers and is suitable for assessment for health risks. Neither resveratrol nor irbesartan affected the plasma cotinine level. Thus it is likely that similar nicotine dosing was administered to all the rats. Nonetheless, future studies should take into account of measuring cotinine weekly together with other nicotine metabolites to understand whether there is an association between nicotine metabolism and kidney injury progression.

Hypertension is also an important risk factor for developing kidney diseases. High blood pressure is associated with renal oxidative stress, glomerular injury, microalbuminuria and renal fibrosis [45]. In this study, 28 days of nicotine administration resulted in a significant increment in systolic blood pressure, which was effectively blunted by the resveratrol co-administration. Resveratrol was reported among several past studies to exhibit remarkable blood pressure lowering activity in spontaneously hypertensive rats and mouse models of hypertension via its antioxidant activity, vascular protection and nitric oxide production [46,47,48]. Consistent with these studies, our data suggested that resveratrol exhibit blood pressure lowering activity, which prevented nicotine-induced hypertension in this study. It is likely that anti-hypertensive activity of resveratrol may have also contributed to reduction in kidney damage among nicotine-administered rats, apart from its ROS scavenging effect.

One major limitation of this study is unavailability of data from metabolic cage and urinalysis due to no access to metabolic cages. Urine albumin i.e., microalbuminuria is strong indicator of chronic kidney disease. Unfortunately, due to our technical limitation, we were unable to determine whether nicotine causes microalbuminuria and affects any changes in urinalysis for kidney electrolytes. Future studies therefore are warranted to conduct analysis on urine markers and electrolytes to better understand impact of nicotine intake on kidney dysfunction and damage.

## 5. Conclusions

Taken together, this study showed that resveratrol supplementation prevented nicotine-induced oxidative stress and kidney injury in rat model, comparable to angiotensin II receptor blocker, irbesartan. It is postulated that resveratrol prevented the increase in kidney injury markers through the augmentation of endogenous antioxidant capacity and the inhibition of lipid peroxidation. Resveratrol may serve as an excellent nutraceutical supplement in preventing onset of kidney injury among chronic smokers. Future studies are warranted to elucidate long term benefits of resveratrol relating to characteristics of end-stage kidney diseases such as glomerulosclerosis and renal fibrosis.

## Figures and Tables

**Table 1:** Body weight, systolic blood pressure and cotinine in all experimental groups.

Parameters	Vehicle (*n* = 7)	NIC (*n* = 8)	NIC+R (*n* = 8)	NIC+IRB (*n* = 8)
Body weight (g)	262 ± 18	249 ± 16	255 ± 12	242 ± 21
Systolic blood pressure (mmHg)	113 ± 14	152 ± 17 *	108 ± 11 #	110 ± 13 #
Cotinine (ng/mL)	ND	169 ± 21	142 ± 24	151 ± 32

ND, not detected. * *p* < 0.05 vs. vehicle and # *p* < 0.05 vs. NIC using one-way ANOVA with Tukey post-hoc.
